# Moesin is involved in microglial activation accompanying morphological changes and reorganization of the actin cytoskeleton

**DOI:** 10.1186/s12576-020-00779-6

**Published:** 2020-10-31

**Authors:** Tomonori Okazaki, Daichi Saito, Masatoshi Inden, Kotoku Kawaguchi, Sayuri Wakimoto, Takashi Nakahari, Shinji Asano

**Affiliations:** 1 Department of Molecular Physiology, College of Pharmaceutical Sciences, Ritsumeikan University, 1-1-1 Noji-Higashi, Kusatsu, 525-8577 Japan; 2 Laboratory of Medical Therapeutics and Molecular Therapeutics, Gifu Pharmaceutical University, 1-25-4 Gifu City University Nishi, Gifu, 501-1196 Japan; 3 Research Unit for Epithelial Physiology, Research Organization of Science and Technology, Ritsumeikan University, 1-1-1 Noji-Higashi, Kusatsu, 525-8577 Japan

**Keywords:** Microglia, Moesin, Actin cytoskeleton, Phagocytosis

## Abstract

Moesin is a member of the ezrin, radixin and moesin (ERM) proteins that are involved in the formation and/or maintenance of cortical actin organization through their cross-linking activity between actin filaments and proteins located on the plasma membranes as well as through regulation of small GTPase activities. Microglia, immune cells in the central nervous system, show dynamic reorganization of the actin cytoskeleton in their process elongation and retraction as well as phagocytosis and migration. In microglia, moesin is the predominant ERM protein. Here, we show that microglial activation after systemic lipopolysaccharide application is partly inhibited in moesin knockout (Msn-KO) mice. We prepared primary microglia from wild-type and Msn-KO mice, and studied them to compare their phenotypes accompanying morphological changes and reorganization of the actin cytoskeleton induced by UDP-stimulated phagocytosis and ADP-stimulated migration. The Msn-KO microglia showed higher phagocytotic activity in the absence of UDP, which was not further increased by the treatment with UDP. They also exhibited decreased ADP-stimulated migration activities compared with the wild-type microglia. However, the Msn-KO microglia retained their ability to secrete tumor necrosis factor α and nitric oxide in response to lipopolysaccharide.

## Introduction

Microglia are immune cells resident in the central nervous system. Under normal or resting conditions, microglia with numerous long and branched processes continuously survey their environment to identify abnormalities in surrounding cells. In responding to injury, they retract their processes, induce hypertrophy of their cell bodies, and rapidly migrate toward the site of injury [1]. They secrete a number of proinflammatory cytokines, including tumor necrosis factor α (TNF-α), interleukin-1β, and cytotoxic molecules such as nitric oxide (NO) and reactive oxygen species, [1] but also neuroprotective molecules such as interleukin-10 and TGF-β [2]. They are also engaged in the clearance of dead and dying neurons and neuronal debris by phagocytosis, which is crucial to the maintenance of brain functions [2]. Such process elongation and retraction and phagocytosis are driven by dynamic reorganization of the actin cytoskeleton [3].

Microglia contain several metabotropic P2Y (P2Y_6_, P2Y_12_, and P2Y_13_) and ionotropic P2X (P2X_4_ and P2X_7_) receptors, some of which are involved in phagocytosis, migration and chemotaxis [4]. Among them, microglia express the metabotropic P2Y_6_ receptor whose activation by endogenous agonist UDP triggers phagocytosis [5]. The activation of the purinergic P2Y_6_ receptor with UDP evokes microglia motility through the phosphorylation of an actin-binding vasodilator-stimulated phosphoprotein (VASP), which results in phagocytosis [6]. VASP phosphorylation is dependent on Rho, which is regulated by ezrin, radixin and moesin (ERM) proteins. Microglia also express the metabotropic P2Y_12_ and the ionotropic P2X_4_ receptors whose activation by the endogenous agonist ATP triggers chemotaxis [7]. The activation of purinergic P2Y_12_ receptor with ATP evokes microglia motility through the activation of phosphatidylinositol 3′-kinase (PI3K) and phosphorylation of protein kinase B (Akt). Rac GTPase, which may interact with ERM proteins, regulates a positive feedback loop between PI3K and actin polymerization [8].

Moesin is a member of ERM proteins, which are involved in the formation and/or maintenance of cortical actin organization through their cross-linking activity between actin filaments and proteins and/or phospholipid located on the plasma membranes [9]. ERM proteins also regulate the function of small GTPases including the activities of Rac, Rho, and Cdc42 [9, 10]. Consequently, ERM proteins in general play important roles in the formation of microvilli, filopodia, uropods, and ruffling membranes where actin filaments are associated with plasma membranes [11]. They are also directly involved in growth cone morphology, motility, and process formation in primary cultured neurons [12]. The function of ERM proteins seems to be redundant and complementary with one another in vitro.

Moesin was found to be very important for the physiological regulation of neutrophils, lymphocytes, and renal tubule cells based on investigations employing moesin knockout (Msn-KO) mice. Neutrophils are able to adopt a polarized morphology, migrate and phagocytose pathogens. In the neutrophil, moesin controls polarization and chemotaxis through interactions with small G proteins [13]. Consequently, Msn-KO mice exhibited decreased bacterial clearance and inflammation. As for lymphocytes, egress of T and B cells from the thymus and bone marrow is regulated by sphingosine 1-phosphate (S1P) and its receptor on the surface of cells. Moesin regulates clathrin-dependent S1P receptor 1 (S1PR1) internalization, and thus the egress of T and B cells [14, 15]. In fact, Msn-KO mice exhibited decreases in B and T cells in the peripheral blood and lymph nodes. Moesin also binds with the Na^+^, K^+^, 2Cl^−^ transporter 2 (NKCC2) and regulates its endocytosis in the tubules of thick ascending limb of Henle [16].

Moesin is the predominant ERM protein in microglia. Microglia exhibit dynamic reorganization of the actin cytoskeleton as mentioned above. However, there has been no report on the role of moesin in microglia to date. Here, we studied the roles of moesin on the microglial activation after systemic lipopolysaccharide (LPS) application in vivo by using the wild-type (WT) and Msn-KO mice. We also studied the roles of moesin in microglial morphology, phagocytosis, migration, and production and secretion of TNF-α and NO in vitro by comparing the phenotypes of primary cultured microglia prepared from the WT and Msn-KO mice.

## Materials and methods

### Experimental animals and treatment

Mice were acclimated to and maintained at 23˚C under a 12 h light/dark cycle (light on 0800 am–2000 pm) and fasted overnight with free access to water. All animal experiments were carried out in accordance with the National Institutes of Health Guide for the Care and Use of Laboratory Animals, and the protocols were approved by the Animal Ethics Committees of Ritsumeikan University. Msn-KO (*Msn*^−/Y^) mice were prepared previously, and were the kind gifts of Professor S. Tsukita of the Graduate School of Frontier Biosciences, Osaka University [17].

Genotyping of mice was performed by PCR of mouse tail genomic DNA, by using a combination of primers specific for the wild-type allele and for the targeted allele. The forward and reverse primers for the wild-type allele were 5′-CTGAAGTCGGACAAAGATTTCCAGG-3′ and 5′-AGGTGTCTCCCAGA GATACGATTTGG-3′, respectively. The forward primer for the targeted allele was 5′-CATCAGTATATG AAACAGCCCCCTG-3’.

In the in vivo experiment, 3-month-old mice were injected intraperitoneally on four consecutive days with 100 µL LPS (1 µg LPS/g body weight) or 100 µL PBS vehicle as control and the immunohistochemical analysis was performed on the fifth day since the start of the treatment as reported previously [18].

### Antibodies and reagents

Anti-ionized calcium-binding adapter molecule (Iba1) antibody (rabbit) (019–19741) was from FUJIFILM Wako Pure Chemical (Osaka, Japan). Anti-moesin antibody (mouse) (2287) was a kind gift from Prof. Tsukita. Anti-ERM antibody (rabbit) (3142) and anti-iNOS (2982) antibody (rabbit) were from Cell Signaling Technologies (Danvers, MA. USA). Alexa Fluor 488-conjugated goat anti-rabbit IgG (H + L) (A11008) and Alexa Fluor 633-conjugated goat anti-mouse IgG secondary antibodies (A21050) were from Thermo Fisher Scientific (Waltham, MA, USA).

Rhodamine phalloidin (R415) was purchased from Thermo Fisher Scientific (Waltham, MA, USA). NO_2_/NO_3_ Assay Kit-CII and fura-2-acetoxymethyl ester (Fura-2 AM) were purchased from Dojindo Laboratories (Kumamoto, Japan). Fluoresbrite carboxylate microspheres fluorescent beads (6 µm) was purchased from Polysciences Inc. (Warrington, PA, USA). LPS from *E. coli* O111:B4 was purchased from Merck (Darmstadt, Germany). Mouse TNF-α Quantikine ELISA Kit was purchased from R&D Systems (Minneapolis, MN, USA).

### Tissue preparation and immunohistochemistry

Treated mice were perfused under deep anesthesia with pentobarbital (100 mg/kg, i.p.). Briefly, after perfusion, the brain was quickly removed and postfixed. The brain slices were cut into 20-µm-thick sections using a cryostat and collected in 100 mM PBS containing 0.3% Triton X-100 (PBS-T). After several washes, the slices were stored until use in free-floating state at 4˚C for immunohistochemical analysis. For immunofluorescence staining, brain slices were incubated with primary antibody, including Iba1 (1:3,000 dilution) for 3 days at 4 ˚C. The antibody was detected by Alexa488-conjugated secondary antibody (1:500 dilution). Fluorescence was observed using a laser scanning confocal microscope (LSM700, Zeiss). Semi-quantitative image-analysis of immunopositive cells was performed in the middle of the substantia nigra pars compacta (SNpc), as described previously [19].

### Preparation of primary cultured microglia

Primary cultured microglia were prepared from newborn WT and Msn-KO mice [20]. Briefly, mice at postnatal day 1 were anesthetized by hypothermia, and the brains were removed from the skull and minced with scissors. Tissue fragments were incubated at 37 °C for 15 min in 0.25% trypsin (Nacalai Tesque, Kyoto, Japan) containing 0.1 mg/ml bovine pancreatic DNaseI (Sigma-Aldrich, St. Louis, MS, USA). Following centrifugation at 1700 rpm for 15 min, the supernatant was discarded, and the pellet was re-suspended in DMEM supplemented with 10% FBS, 5 μg/ml insulin (FUJIFILM Wako Pure Chemical, Osaka, Japan), and 100 units/mL penicillin G and 100 μg/mL streptomycin. The cells were seeded on 75-cm^2^ flasks and maintained at 37 °C in a humidified 5% CO_2_ atmosphere. The medium was changed 2 days after seeding and then every 3 or 4 days. After 2–3 weeks, the primary mixed glial cultures in flasks were shaken for 90 min at 150 rpm. The detached cells were plated on plastic dishes. Usually, cultures were found to contain 90% microglia by immunostaining with an antibody against Iba1.

### Immunofluorescence analysis of primary cultured microglia

Microglia on glass coverslips coated with poly-D-lysine were fixed in 4% paraformaldehyde at room temperature for 15 min and washed three times with PBS containing 10 mM glycine. Cells were permeabilized in a permeabilization buffer (PBS containing 0.1% Triton X-100) at room temperature for 5 min. The coverslip was blocked by pre-incubating in PBS containing 3% BSA at room temperature for 10 min. All antibody incubations were carried out using Can Get Signal Immunostain Solution A (Toyobo, Osaka, Japan). The coverslip was incubated overnight at 4℃ with anti-Iba1 (1:100 dilution) and anti-moesin (1:10 dilution) antibodies, followed by three washes with PBS containing 0.1% BSA. Alexa Fluor 488 goat anti-rabbit IgG (H + L) (A11008, 1:100 dilution), Alexa Fluor 633-conjugated goat anti-mouse IgG secondary antibodies (A21050, 1:100 dilution) and Rhodamine phalloidin (R415, 1:100 dilution) were incubated with the cells on the coverslip for 30 min at room temperature. After a final PBS wash, the coverslips were mounted with fluorescent mounting medium (Funakoshi, H-1000, Japan) and examined using a confocal laser scanning microscope (FV-10i, Olympus, Tokyo, Japan).

### Membrane ruffling of primary cultured microglia

Membrane ruffling was examined as described previously [21]. Microglia were attached to glass coverslips coated with poly-D-lysine. After attachment for 2 h, microglia were washed with serum-free DMEM and starved for 4 h in the same medium. They were stimulated with 50 µM ADP or UDP at for 5 min at 37 ℃. After fixation with 4% paraformaldehyde for 5 min and rinsed with PBS, the cells were permeabilized with PBS containing 0.1% Triton X-100 for 5 min and washed three times with PBS. Cells were stained with rhodamine phalloidin and examined with the confocal laser scanning microscope.

### Phagocytosis assay

Phagocytosis was examined with Fluoresbrite carboxylate microspheres beads as described previously with some modification [22]. Microglial cells (2 × 10^5^ cells) were cultured on poly-D-lysine-coated glass coverslip in 24-well plates and incubated with serum-free DMEM for 1 h. The cells were then incubated with the FBS-coated Fluoresbrite carboxylate microspheres fluorescent beads (6 μm diameter) (1:125 diluted) in serum-free DMEM at 37℃ for 45 min with or without 10 μM UDP. The cells were thoroughly washed with cold PBS to remove free beads. The cells were fixed in 4% paraformaldehyde for 15 min and permeabilized with PBS containing 0.1% Triton X-100 for 5 min at room temperature. The cells were stained with an anti-Iba1 antibody as well as rhodamine phalloidin. The number of fluorescent microspheres incorporated in microglia was counted under the fluorescent microscope and the confocal laser scanning microscope.

### Migration assay

Migration was examined in Boyden chambers as described previously [21, 23]. In short, primary microglia (5 × 10^4^ cells/well) suspended in serum-free DMEM were plated on Costar Transwell® plates (6.5 mm diameter insert, 8.0 μm pore size, polycarbonate membrane filter; Corning Inc.), and allowed to migrate for 2 h at 37℃. The filter was coated with 10 μg/ml fibronectin beforehand. The bottom chamber of these plates contained either 0 or 10 μM ADP. Following migration, the medium in the top chamber was removed and the cells on the filter were fixed with 3.7% formaldehyde in PBS for 2 min. The filter was rinsed twice with PBS and treated with methanol for 20 min. The filter was then rinsed twice with PBS, followed by staining with Giemsa solution (079–04391, FUJIFILM Wako Pure Chemical, Osaka, Japan). After two more rinses with PBS, the filter was wiped with a cotton swab to remove the cells that did not migrate. Cells that migrated across the filter in six random fields were counted for each condition using phase contrast microscopy. Each experiment was repeated at least three times. Results are expressed as mean cell migration ± SEM.

### Ca^2+^ measurements

Intracellular Ca^2+^ concentration ([Ca^2+^]_i_) was estimated by the measurement of Ca^2+^-sensitive fluorescent dye, Fura-2 AM as described previously [24]. Cells were set on a coverslip precoated with Cell-Tak (Becton Dickinson Labware, Bedford, MA, USA) in Krebs–Ringer buffer (140 mM NaCl, 5 mM KCl, 1 mM MgCl_2_, 2 mM CaCl_2_, 10 mM HEPES, 10 mM glucose, pH 7.4) containing 5 μM Fura-2 AM for 30 min at room temperature. The cells were rinsed with the buffer, and the coverslips were transferred to a microperfusion chamber on the stage of an inverted fluorescence microscope. Fluorescence signal was obtained with excitation wavelength of 340/380 nm, emission wavelength of 510 nm. [Ca^2+^]_i_ was monitored as the ratio of fluorescence intensity of Fura-2 at 340 nm and 380 nm.

### NO production

Primary microglia (4 × 10^5^ cells/well) suspended in DMEM containing 10% FBS were seeded in 24-well plates, and incubated with 1 μg/ml LPS for 24 h at 37 ℃. Supernatants were collected from 24-well plates and assessed for NO production using the NO_2_/NO_3_ Assay Kit-C II based on Griess assay following the manufacturer’s protocol. All treatments were completed at least three times and data were expressed as mean ± SEM.

### Western blot

Primary microglia in 24-well plates were washed with phosphate-buffered saline, homogenized with the lysis buffer (50 mM Tris–HCl, pH 7.4, 150 mM NaCl, 0.5 mM EDTA, 1% Nonidet-P40 with protease inhibitor cocktail) and centrifuged at 16,000× *g* for 20 min. The supernatant was used as cell lysate. Proteins were separated by Laemmli’s sodium dodecyl sulfate–polyacrylamide gel electrophoresis (8–12.5%), and then transferred to a polyvinylidene difluoride membrane. The membrane was blocked for 1 h with 2.5% milk powder in TBST (10 mM Tris–HCl, pH 8.5, 150 mM NaCl, and 0.1% Tween 20), and exposed to primary antibodies diluted with Solution 1 (*Can Get Signal*® Immunoreaction Enhancer Solution, TOYOBO) overnight at 4 °C. After rinsing with TBST, the membrane was incubated with secondary antibody diluted with Solution 2 (*Can Get Signal*® Immunoreaction Enhancer Solution, TOYOBO) for 1 h at room temperature. After washing, antigen–antibody complexes on the membrane were visualized with a chemiluminescence system (ECL plus; GE Healthcare, Waukesha, WI).

#### ELISA

Primary microglia (5 × 10^4^ cells/well) suspended in DMEM containing 10% FBS were seeded in 96-well plates, and incubated with 1 μg/ml LPS for 24 h at 37 ℃. Supernatants were collected and assayed for TNF-α production using the Mouse TNF-α Quantikine ELISA Kit (R&D Systems, Minneapolis, MN, USA) following the manufacturer’s protocol.

### Statistical analysis

Statistical analysis was performed by Student’s T test or one-way ANOVA with Turkey’s post hoc test. The confidence limit of *p* < 0.05 was considered to be statistically significant. The results are expressed as the means ± SEM.

## Results

### Microglial activation after systemic LPS application

**Fig. 1:**
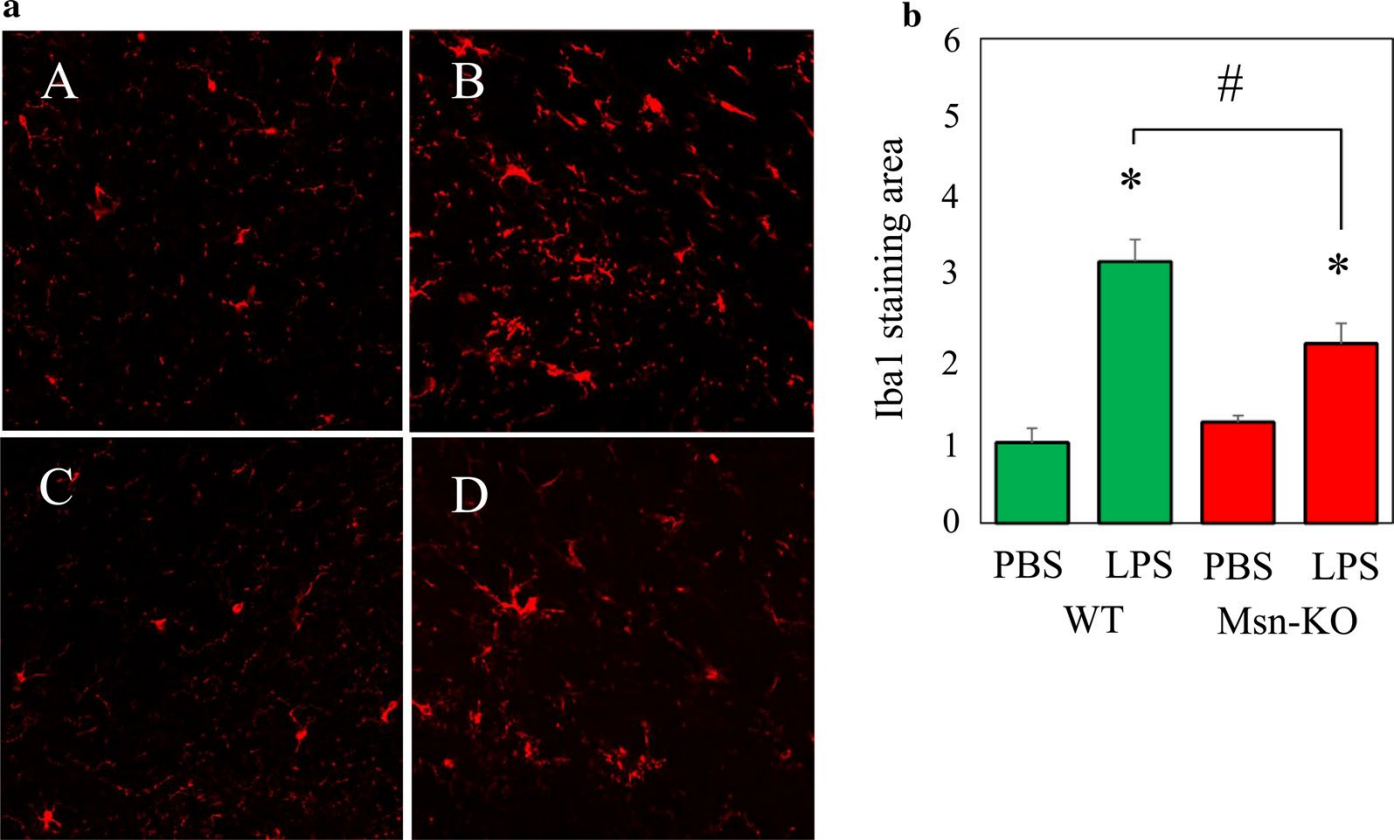
Prolonged microglial activation of the WT and Msn-KO mice after repeated systemic challenge with LPS. **a** Immunofluorescence staining of the microglial marker Iba1 in the WT (**a**, **b**) and Msn-KO (**c**, **d**) mouse SNpc on experimental day 5 after repeated treatment with PBS control (**a**, **c**) or LPS (**b**, **d**). Representative images of 6 mice are shown. **b** Quantification of Iba1 staining area shown in **a** was expressed as the ratio with the value of WT treated with PBS being 1. Results are shown as mean ± SEM. *p < 0.05 vs. PBS, and #*p* < 0.05 vs. WT treated with PBS (*n* = 6 animals)

Detrimental changes in brain parenchyma are quickly sensed by microglial cells [25]. In order to study microglial activation after systemic LPS application to the WT and Msn-KO mice, we analyzed brain tissue on day 5 after systemic repeated application of LPS or PBS vehicle control (Fig. 1a). We studied the microglial Iba1 immunoreactivity in the SNpc, a brain region characterized by a relative high density of microglial cells [26, 27]. In the WT mouse brain, Iba1 staining area was 3.2 times higher in the LPS-treated mice compared with control (PBS-treated) mice. On the other hand, in the Msn-KO mouse brain, Iba1 staining area was 1.8 times higher in the LPS-treated mice compared with control (PBS-treated) mice, which was significantly lower compared with the LPS-treated WT mice (Fig. 1b).

### Expression of ERM proteins

**Fig. 2:**
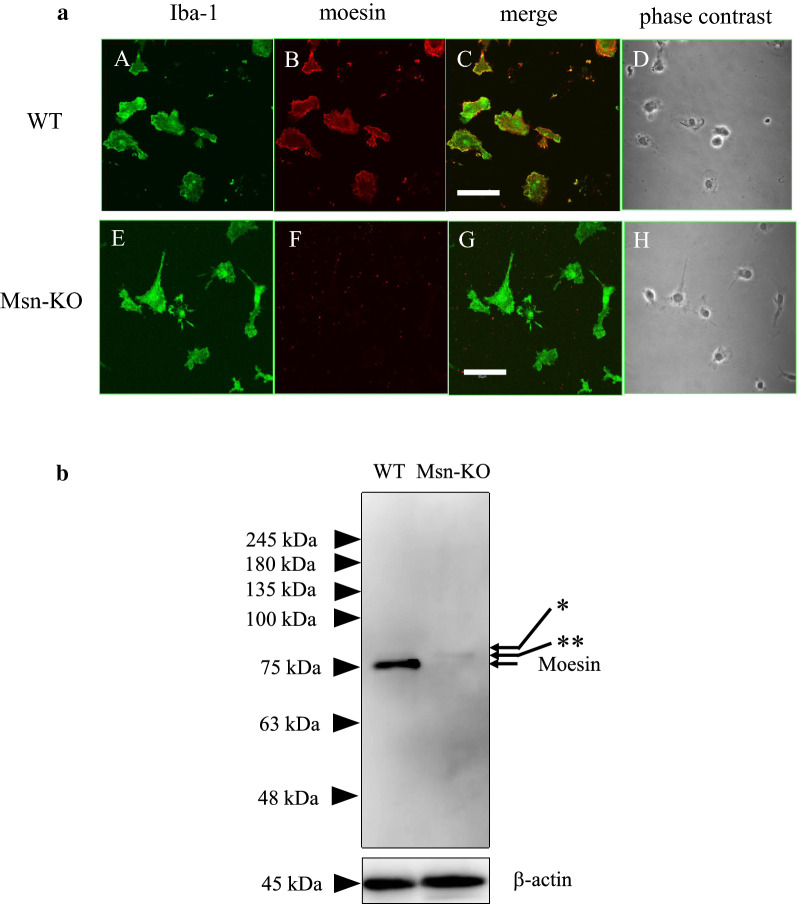
Expression of ERM proteins in primary microglia. **a** The expression of Iba1 (**a** and **e**, in green) and moesin (**b** and **f**, in red) and their merged pattern (**c** and **g**) in the WT (**a**, **b**, **c**) and Msn-KO microglia (**e**, **f**, **g**) was studied by immunofluorescence. Their phase contrast images were also shown (**d**, **h**). Scale bars, 40 μm. **b** The cell lysates of WT and Msn-KO microglia were blotted with an antibody that recognizes all three ERM proteins and with an antibody that detects β-actin (bottom). (Left) The WT-microglia abundantly expressed moesin with a molecular mass of 75 kDa, whereas they expressed very small amounts of ezrin (*) and radixin (**). (Right) The Msn-KO microglia lose moesin without apparent compensatory upregulation of ezrin and radixin

In order to study the roles of moesin on the microglial activation, we examined to compare the functions of primary microglia prepared from WT and Msn-KO mice. At first, we studied the expression of moesin in the WT and Msn-KO microglia. In immunofluorescence analysis, moesin was co-localized with Iba1 in the WT primary microglia as shown in Fig. 2a. The expression of ERM proteins in the primary microglia was studied by using an anti-ERM antibody that recognizes all three ERM proteins in the western blot. As shown in Fig. 2b, the WT-microglia abundantly expressed moesin, whereas they expressed very small amounts of ezrin and radixin. The Msn-KO microglia lost moesin expression without any apparent compensatory upregulation of ezrin and radixin as shown in Fig. 2b.

### Morphology

**Fig. 3:**
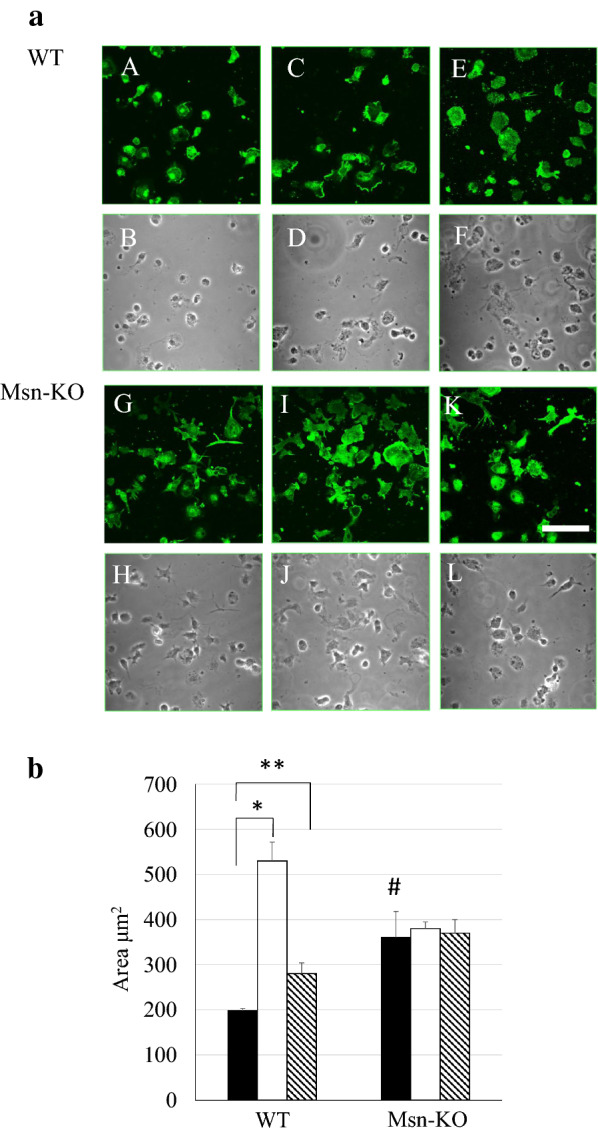
**a** Confocal microscopic images of primary microglia stained with an anti-Iba1 antibody. **a** The WT (**a**, **b**, **c**) and Msn-KO (**g**, **h**, **i**) microglia were treated with serum-free DMEM (as a control) (**a**, **g**), 50 μM ADP (**b**, **h**) or UDP (**c**, **i**) for 5 min at 37℃, and stained with an anti-Iba1 antibody. **b** Their phase contrast images were also shown (**d**, **e**, **f**, and **j**, **k**, **l**). **b** The area stained with anti-Iba1 antibody was calculated with ImageJ software. Closed, open, and hatched columns represent serum-free DMEM, ADP and UDP treatment, respectively. Results are shown as mean ± SEM. * *p* < 0.01 vs. WT treated with serum-free DMEM (*n* = 3), ** *p* < 0.05 vs. WT treated with serum-free DMEM (*n* = 3), and # *p* < 0.05 vs. WT treated with serum-free DMEM (*n* = 3)

It is generally accepted that microglia in primary culture exhibit morphology different from microglia in the brain. They do not possess their characteristic long processes in the resting state. However, they show morphological changes from the resting shape and acquire an activated shape following treatment with ATP, ADP, UTP as well as with LPS and β-amyloid peptide [21, 28]. Figure 3a shows the morphological changes produced in WT and Msn-KO microglia that were stained with the anti-Iba1 antibody following incubation in the presence and absence of ADP and UDP. In the absence of ADP and UDP, WT-microglia showed heterogenous shapes with small cell volume ranging from spindle to round cells. Some of them contained a small number of short processes. They were induced to spread and increased their cell volume with some membrane ruffles in response to the treatment with 50 μM ADP or UDP as reported previously [21]. On the other hand, Msn-KO microglia possessed larger cytoplasmic volumes as well as a number of processes even in the absence of ADP nor UDP. They were further spread and increased their cell volume following treatment with 50 μM ADP or UDP. In Fig. 3b, we quantified the cell volume by measuring the area stained with the Iba1 antibody. In the WT-microglia, the Iba1-positive areas were significantly increased by the treatment with 50 μM ADP or UDP. The Iba1-positive areas were significantly higher in the Msn-KO microglia than the WT-microglia. In the Msn-KO microglia, the Iba1-positive areas were not increased by the treatment with 50 μM ADP or UDP.

**Fig. 4:**
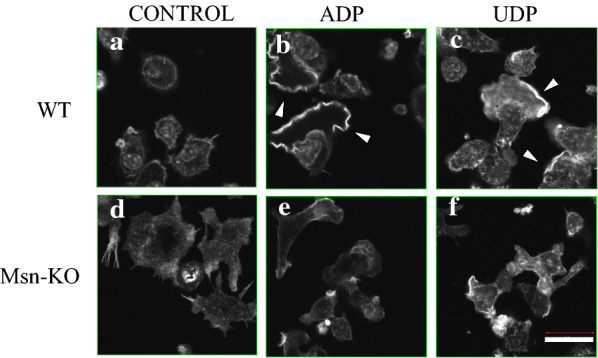
Membrane ruffling in microglia. The WT (**a**, **b**, **c**) and Msn-KO microglia (**d**, **e**, **f**) were treated with serum-free DMEM (as a control) (**a**, **d**), 50 μM ADP (**b**, **e**) or UDP (**c**, **f**) for 5 min at 37℃. After fixation, the cells were permeabilized with PBS containing 0.1% Triton X-100, and stained with rhodamine phalloidin to observe ruffle membranes (shown by arrow heads)

Primary microglia show membrane ruffling which involves a meshwork of newly polymerized actin filaments after treatment with ATP, ADP or UTP [21]. Here, we compared the formation of membrane ruffling of the WT and Msn-KO microglia by staining with phalloidin. The WT-microglia showed membrane ruffling induced by the treatment with ADP or UDP. A small amount of membrane ruffling was observed in the Msn-KO microglia even in the absence of ADP or UDP, which was increased by the treatment of ADP or UDP (Fig. 4).

### Phagocytosis assay

**Fig. 5:**
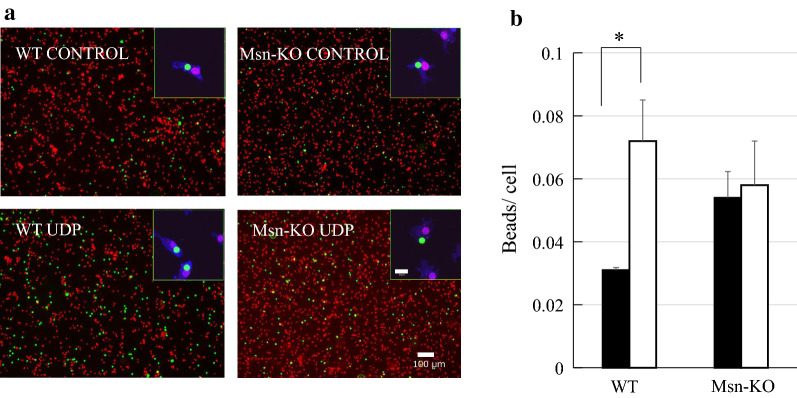
Phagocytosis of fluorescent beads by the WT and Msn-KO microglia following treatment with 10 μM UDP. **a** Representative images of the WT and Msn-KO microglia are shown. Fluorescent beads are shown in green, and nuclei labeled with propidium iodide (PI) are depicted in red. Scale bar, 40 μm. High magnification images obtained by confocal microscopy are presented in the insets, in which fluorescent beads, Iba1 and PI were shown in green, blue and red, respectively. **b** The number of fluorescent beads per WT and Msn-KO microglia was counted in the presence and absence of 10 μM UDP, and shown by closed (without UDP) and open columns (with UDP), respectively. 150–200 cells were studied in each experiment. Results are shown as mean ± SEM. *P < 0.05 vs. WT (*n* = 3 trials)

Phagocytosis is one of the most important physiological functions of microglia and is the process that mediates the uptake of larger particles by actin-based mechanisms. Microglia express the metabotropic P2Y_6_ receptor whose activation by endogenous agonist UDP triggers phagocytosis [5]. In order to study the role of actin-binding protein, moesin in this process, we compared the phagocytotic activity of WT and Msn-KO microglia. The number of fluorescent microbeads taken up by the WT and Msn-KO microglia in the presence and absence of 10 μM UDP was counted using a confocal microscope. Figure 5a shows typical patterns of WT and Msn-KO microglia with incorporated fluorescent microbeads. Figure 5b shows the number of fluorescent beads per cells of the WT and Msn-KO microglia in the presence and absence of 10 μM UDP. Even in the absence of UDP, a small amount of bead was incorporated in the WT-microglia. This basal incorporation of beads decreased to almost zero level at 4℃ (data not shown). The number of beads incorporated in the WT-microglia was increased 2.5-times by the treatment with 10 μM UDP, indicating that the phagocytotic activity was stimulated by UDP. On the other hand, the number of beads incorporated in the Msn-KO microglia was high even in the absence of UDP, which was not further increased by the treatment of UDP. These results suggest that phagocytotic activity of Msn-KO microglia may be active even in the absence of UDP.

### Migration

**Fig. 6:**
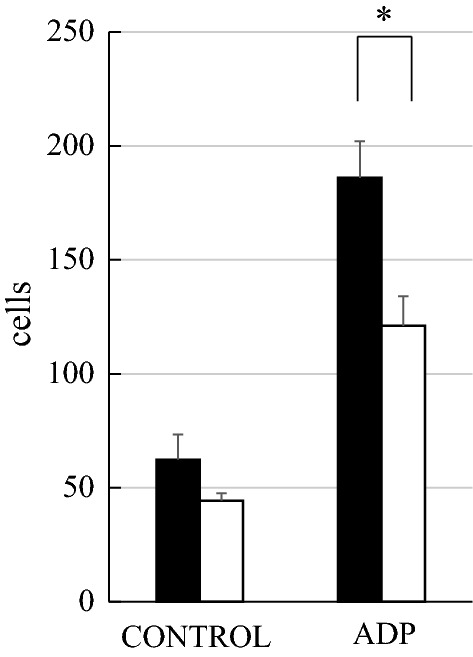
Migration of primary microglia toward ADP was measured. Primary microglia from WT and Msn-KO mice were allowed to migrate towards 0 (control medium) or 10 μM ADP for 2 h in the Boyden chamber, and the number of migrated cells was counted. The numbers of migrated WT and Msn-KO microglia are shown with closed and open columns, respectively. Results are shown as mean ± SEM. WT (*N* = 4), Msn-KO (*N* = 3). *P < 0.05 vs. WT

Extracellular ATP and ADP induce membrane ruffling and cell migration behavior consistent with chemo-attraction, and these effects are mediated by the P2Y_12_ receptor [21]. Figure 6 shows the numbers of WT and Msn-KO microglia that migrated toward serum-free DMEM (control) and ADP in the Boyden chamber. The number of migrated cells increased in the presence of ADP compared with the control conditions both for the WT and Msn-KO microglia. However, the number of migrated cells was significantly smaller in the Msn-KO than the WT-microglia. These results suggest that moesin is not indispensable, but is nonetheless important for the regulation of migration.

### Ca^2+^ imaging

**Fig. 7:**
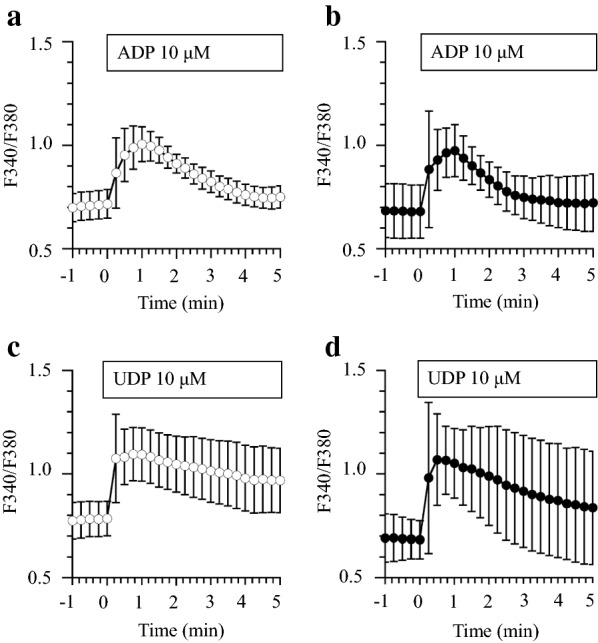
ADP- and UDP-induced calcium response of primary microglia. Primary microglia from WT (**a, c**) and Msn-KO mice (**b, d**) loaded with 5 μM Fura-2 AM were stimulated with 10 μM ADP (**a, b**) or UDP (**c, d**), respectively, at the zero minute time point. Intracellular calcium levels are expressed as the F340/F380 ratio. Results are expressed as mean value ± SEM. WT treated with ADP (*N* = 11), UDP (*N* = 19). Msn-KO treated with ADP (*N* = 13), UDP (*N* = 5)

Microglia express metabotropic P2Y_12_ and P2Y_6_ receptors that are coupled to the activation of phospholipase C, leading to the production of inositol 1,4,5-triphosphate (InsP3) and the release of Ca^2+^ from InsP3-receptor-sensitive stores [5, 29]. Here, we monitored the change of [Ca^2+^]_i_ in the WT and Msn-KO microglia during stimulation with ADP (10 µM) and UDP (10 µM). In the WT and Msn-KO microglia, ADP evoked a transient increase followed by a sustained increase in [Ca^2+^]_i_ as shown in Fig. 7a and b. The levels of sustained increase returned to those observed before the start of ADP stimulation. Values of the transient peak in the Msn-KO microglia were similar to those in the WT-microglia. UDP also evoked a transient increase followed by a sustained increase in [Ca^2+^]_i_ of the WT and Msn-KO microglia as shown in Fig. 7c and d. However, levels of the sustained [Ca^2+^]_i_ increase stimulated by 10 µM UDP were much higher than those stimulated with 10 µM ADP. The peak [Ca^2+^]_i_ values in the Msn-KO microglia were also almost similar to those in the WT-microglia during UDP stimulation. Thus, [Ca^2+^]_i_ increases stimulated by ADP and UDP in the Msn-KO microglia are similar to those in WT-microglia. These results suggest that moesin is not involved in Ca^2+^ signaling stimulated by ADP-binding to P2Y_12_ or UDP-binding to P2Y_6_ receptor.

### NO production and iNOS expression

**Fig. 8:**
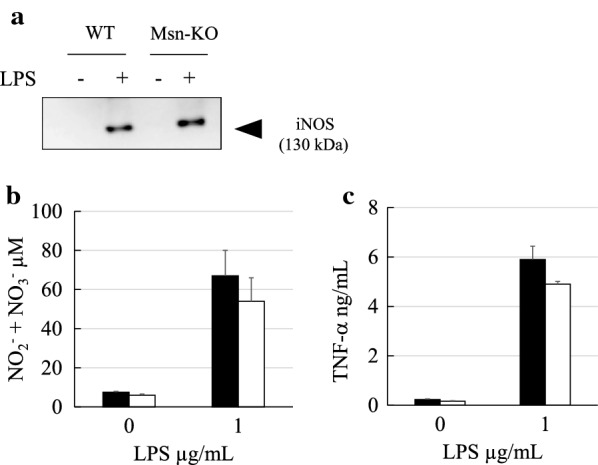
NO and TNF-α secretion by primary microglia is upregulated by treatment with LPS. **a** The cell lysates of WT and Msn-KO microglia treated with or without LPS were blotted with an anti-iNOS antibody. Expression of iNOS was upregulated by the treatment with LPS in the WT and Msn-KO microglia. **b** NO release from the WT and Msn-KO microglia treated with or without LPS was measured by Griess assay, and shown by closed and open columns, respectively. NO release was upregulated by the treatment with LPS in the WT and Msn-KO microglia. **c** TNF-α release from the WT and Msn-KO microglia treated with or without LPS was measured by ELISA, and shown by closed and open columns, respectively. TNF-α release was upregulated by the treatment with LPS in the WT and Msn-KO microglia. Results are expressed as mean value ± SEM. WT (*N* = 7), Msn-KO (*N* = 4). *p < 0.05 vs. control

Primary microglia stimulated with LPS secrete proinflammatory cytokines. Toll-like receptor 4 (TLR4) is involved in this process [30]. Primary microglia are known to upregulate mRNA and protein expression of iNOS in response to treatment with LPS [31]. In fact, the band representing iNOS with a molecular mass of 135 kDa was not observed in the absence of LPS, whereas it was observed in the presence of 1 μg/ml LPS by the western blot as shown in Fig. 8a. The expression of iNOS protein was also upregulated by LPS in the Msn-KO microglia, and its expression level was almost comparable between the WT and MKO microglia (Fig. 8a). The level of NO secretion by the WT-microglia was tenfold increased by the treatment with 1 μg/ml LPS, which was consistent with upregulation of iNOS protein by LPS (Fig. 8b). The level of NO secretion in the presence of 1 μg/ml LPS was almost comparable between the WT and Msn-KO microglia (Fig. 8b).

### TNF-α production

Primary microglia are also known to upregulate mRNA and protein expression of TNF-α following treatment with LPS [31]. The level of TNF-α secretion by the WT-microglia was increased following treatment with 1 μg/ml LPS (Fig. 8c). The level of TNF-α secretion in the presence of 1 μg/ml LPS was almost comparable between the WT and Msn-KO microglia (Fig. 8c).

## Discussion

Microglia show dynamic reorganization of their actin cytoskeleton from the resting ramified to the activated ameboid morphology. Moesin is a member of the ERM protein family whose members are reported to be involved in the formation and/or maintenance of cortical actin organization [9]. They are activated by phosphorylation and also involved in axonal outgrowth, morphological rearrangement, and motility of primary neurons [12, 32, 33]. Among them, moesin is involved in polarization and chemotaxis through interactions with small G proteins, Rac, Rho, and Cdc-42 in neutrophils [13]. In resting neutrophils, moesin is activated and prevents cell polarization by inhibiting the small GTPases, whereas it is deactivated by attractant-sensitive myosin phosphatase to break symmetry and establish polarity. In lymphocytes, moesin is involved in regulating clathrin-dependent S1P receptor 1 (S1PR1) internalization [15]. Msn-KO mice exhibited decreased numbers of T and B cells in the blood due to the impairment of their egress from thymus and bone marrow, respectively. Moesin is the predominant ERM protein in microglia which express very low levels of ezrin and radixin as shown in Fig. 2b. Therefore, we explored the possibility that moesin is involved in morphological changes, phagocytosis and migration processes, which follow actin reorganization. Towards this end, we studied microglial activation in the SNpc after systemic LPS application in vivo in the WT and Msn-KO mice and found that the activation is partly inhibited in the Msn-KO mice.

Primary microglia in vitro do not have the ramified structure typically seen in the normal brain. They show heterogeneous shapes, ranging from spindle and rod-shaped at one extreme, to amoeboid versions with short thick processes expanding as lamellipodia or round cells at the other extreme [1]. However, many of the phenotypes associated with microglia in situ can be observed in the primary microglia. They migrate toward ADP and ATP, which are signaling factors (“find-us” signal) released from damaged or dead cells. They phagocytose particles in the presence of UDP, which is also released from damaged or dead cells as the “eat-us” signal [34]. They also secrete TNF-α, interleukin-1β, and NO into the media following treatment with LPS. Therefore, we further compared the phenotypes exhibited by WT and Msn-KO primary microglia.

The WT primary microglia showed morphological changes in response to treatment with ADP and UTP. They contained modest amounts of cytoplasm and a small number of processes in the absence of ADP and UTP, whereas they formed ruffling membranes, spread, and increased cell volume following treatment with ADP or UDP (Fig. 3b). However, the Msn-KO microglia contained larger cell volume with a smaller number of ruffling membrane in the absence of ADP or UDP. There was little morphological change of the Msn-KO microglia before and after the treatment with ADP or UDP. Therefore, moesin seems to be involved in determining the morphology of primary microglia and the response of that morphology to activating factors.

Microglia phagocytose particles in response to treatment with UDP. UDP binds to the P2Y_6_ receptor, which is coupled to the activation of phospholipase C, leading to the production of InsP3 and the release of Ca^2+^ from InsP3-receptor-sensitive stores [5]. In fact, UDP treatment produced increases in [Ca^2+^]_i_ in the WT-microglia [5]. There was no significant difference in the [Ca^2+^]_i_ responses elicited by UDP between the WT and MKO microglia (Fig. 7). It was also reported that UDP rapidly induces phosphorylation of one of the actin-binding proteins, VASP in a Rho-dependent manner, and the phosphorylation of VASP results in microglial actin aggregation [6]. In the present study, we showed that the phagocytosis activity of Msn-KO microglia in the absence of UDP was higher than that of WT-microglia, and not further increased by the treatment with UDP (Fig. 5). These results suggest that moesin may negatively regulate phagocytosis. In the Msn-KO microglia, VASP may be phosphorylated in the absence of UDP. These observations suggest that moesin exerts suppressive effects on Rho or on its downstream effectors.

Microglia migrate toward ATP and ADP. ATP and ADP bind to the P2Y_12_ receptor, which is coupled to activation of PI3 kinase (PI3K) and Akt phosphorylation, as well as to activation of phospholipase C and increase in [Ca^2+^]_i_ [29]. Consequently, selective localization of PI3K to the leading edge of the membrane allows the spatially restricted production of phosphatidylinositol 3,4,5-triphosphate (PtdIns(3,4,5)P_3_), which induces F-actin polymerization at the front of migrating cells. Rac GTPase, which may interact with moesin, is known to regulate a positive feedback loop between PI3K and actin polymerization [8]. In the present study, we showed that the migration activity induced by an ADP gradient was retained in the Msn-KO microglia. However, the migration activity of Msn-KO microglia was significantly lower compared with the WT-microglia (Fig. 6). On the other hand, there was no significant differences in the [Ca^2+^]_i_ responses elicited by ADP between the WT and Msn-KO microglia (Fig. 7). These results suggest that moesin is important but not indispensable for migration and chemotaxis.

Microglia are well-known to be activated by LPS through its interaction with the TLR4 receptor. LPS treatment leads to upregulation of the expressions of the iNOS and TNF-α proteins within 24 h. However, in our present study, there was no significant differences in the LPS-stimulated expression in iNOS and TNF-α proteins nor in the secretion of NO and TNF-α between the WT and Msn-KO microglia (Fig. 8). Therefore, moesin is not directly involved in the communication between the TLR4 receptor and upregulation of iNOS and TNF-α genes through nuclear factor-kappa B.

Judging from the results shown above, moesin seems to be involved in microglial activation with morphological changes, although it is difficult to reconcile our results in vivo with in vitro at present.

## Conclusions

In conclusion, moesin is important for microglial activation after systemic LPS application in vivo. It is also important for UDP-induced phagocytosis and ADP-induced migration of primary microglia in vitro. However, moesin is not involved in Ca^2+^ signaling elicited by ADP and UDP. The precise molecular mechanism through which it exerts its effects will be explored more extensively in a future study.

## Data Availability

All data analyzed during this study are included in this published article.

## Abbreviations

ERM: Ezrin, radixin and moesin
Fura-2 AM: Fura-2-acetoxymethyl ester
Iba1: Ionized calcium-binding adapter molecule
LPS: Lipopolysaccharide
Msn-KO: Moesin-knockout
NO: Nitric oxide
PI3K: Phosphatidylinositol 3′-kinase
SFM: Serum-free medium
TNF-α: Tumor necrosis factor α
VASP: Vasodilator-stimulated phosphoprotein
SNpc: Substantia nigra pars compacta
